# *Lactobacillus helveticus* HY7801 ameliorates bacterial vaginosis by inhibiting biofilm formation and epithelial cell adhesion of *Gardnerella vaginalis*

**DOI:** 10.1007/s10068-022-01208-7

**Published:** 2022-12-01

**Authors:** Joo Yun Kim, Eun Chae Moon, Ju-Yeon Kim, Hyeon Ji Kim, Keon Heo, Jae-Jung Shim, Jung-Lyoul Lee

**Affiliations:** R & BD Center, hy Co. Ltd., 22, Giheungdanji-ro 24beon-gil, Giheung-gu, Yongin-si, 17086 Republic of Korea

**Keywords:** Probiotics, Biofilm, Anti-inflammatory, Bacterial vaginosis

## Abstract

Bacterial vaginosis (BV) is caused by a microbial imbalance in the vaginal ecosystem, which causes genital discomfort and a variety of potential complications in women. This study validated the potential of *Lactobacillus helveticus* HY7801 as a probiotic to benefit vaginal health. In vivo, HY7801 reduced the number of *Gardnerella vaginalis* (GV) and pro-inflammatory cytokines in the vagina of GV-induced BV mice and ameliorated vaginal histological changes. In vitro, HY7801 exhibited positive resistance to simulated gastrointestinal conditions, showed excellent adherence ability to the female genital epithelium, and had high lactic acid and H_2_O_2_ production capacity. Furthermore, it was found that HY7801 can alleviate BV because it can suppress the expression of virulence factor genes of GV involved in epithelial cell adhesion and biofilm formation along with antibacterial activity against GV. These results indicate that HY7801 can be used as a promising probiotic strain for the maintenance of a healthy vaginal physiological state.

**Supplementary Information:**

The online version contains supplementary material available at 10.1007/s10068-022-01208-7.

## Introduction

BV is a reproductive disease that occurs frequently in up to 70% of women of childbearing age due to dysbiosis of the vaginal microflora, which includes a decrease in the number of beneficial bacteria such as *Lactobacillus* spp. and increase in the number of harmful bacteria (Muzny et al., 2018; Redelinghuys et al., 2020). BV is not a sexually transmitted infection, but an increase in cytolytic enzymes secreted by pathogenic bacteria such as GV reduces the effect of leukocytes in the host, thus increasing the risk of sexually transmitted infections such as human immunodeficiency virus, human papillomavirus, and pelvic inflammatory disease (Jang et al., 2017; Lewis et al., 2012; Webb, 2021).

GV is an obligate anaerobic bacterium that accounts for most cases of BV and found in approximately 95% of BV patients (Catlin, 1992). GV produces virulence factors such as vaginolysin and sialidase, which are encoded by *vly* and *sld.* It has been reported that vaginolysin from GV acts as a hemolytic toxin and interferes with immune cell activity, and sialidase hydrolyzes immunoglobulin A to promote persistence of BV (Lewis et al., 2012; Qian et al., 2021). In addition, GV is known to interfere with BV antibiotic treatment by attaching to vaginal epithelial cells and forming biofilms that provide a favorable environment for the proliferation of other pathogenic bacteria (Castro and Cerca, 2015; Swidsinski et al., 2011). However, a clear causal relationship between the decrease of *Lactobacillus* spp. in the vagina and the overgrowth of GV has not been elucidated yet (Muzny et al., 2019).

The most common treatment for BV is with antibiotics such as clindamycin and metronidazole (Donders et al., 2014). However, the use of these antibiotics has limitations such as gastrointestinal side effects, an increase in antibiotic-resistant bacteria, and a high recurrence of BV due to a decrease in normal vaginal flora (Donders et al., 2014; Qian et al., 2021). Therefore, recent studies have been actively conducted to alleviate BV and reduce the recurrence rate by using probiotics as substitutes and complements for antibiotics to improve the vaginal environment and suppress the growth of anaerobic pathogens (Huang et al., 2014; Tidbury et al., 2021). Lactic acid bacteria (LAB), the representative probiotics, comprise most of the vaginal flora of healthy women and are known to be effective in preventing BV by producing H_2_O_2_ and lactic acid to suppress the growth of anaerobic pathogens (Boris and Barbes, 2000; Kim et al., 2021). In addition, some LAB strains inhibit pathogenic microorganisms by producing antibacterial peptides such as bacteriocin (Boris and Barbes, 2000; Iseppi et al., 2019; Sabia et al., 2014). However, there is no standard proposed strain as a probiotic for preventing BV (Webb, 2021), and the effect of probiotics on BV may be different even in the same species (Santos et al., 2016; Vásquez et al., 2009). Therefore, ongoing research is needed to determine the optimal probiotic dose and strain for preventing and treating BV.

*Lactobacillus helveticus* HY7801 (HY7801) is a probiotic strain that has been confirmed to alleviate vulvovaginal candidiasis in a previous study. Oral or vaginal administration of HY7801 to animals with vulvovaginal candidiasis reduced pro-inflammatory cytokines and inflammatory enzymes such as COX-2 and inducible nitric oxide synthase. This was found to be due to the antifungal activity of HY7801 against *Candida albicans* and its ability to promote the secretion of anti-inflammatory cytokines such as interleukin-10 (IL-10) (Joo et al., 2012). In this study, we aimed to confirm whether HY7801 could alleviate BV in a GV-infected animal model. In addition, the effect of HY7801 on the growth and biofilm formation of GV was investigated in comparison with the *L. helveticus* type strain. Furthermore, by evaluating the effects of HY7801 on gene expression of relevant parameters including virulence factors, epithelial adhesion, and biofilm formation in GV, we deduced the mechanism by which HY7801 alleviates BV.

## Materials and methods

### Preparation of bacterial strains

*L. helveticus* HY7801 (HY7801) was isolated from healthy Korean woman and stored in the seed culture library at hy Co. Ltd. (Yongin, Republic of Korea). *L. helveticus* ATCC 15009 (ATCC15009), a type strain, was used as a reference strain for the in vitro and in vivo experiments. GV ATCC14018 was obtained from Food Safety Engineering Lab, Seoul National University (Seoul, Republic of Korea). LAB strains (HY7801 and ATCC15009) were cultured in de Man, Rogosa, and Sharpe (MRS) medium (KisanBio, Seoul, Republic of Korea) at 37 °C for 18 h. GV was anaerobically cultured in brain heart infusion (BHI) medium (Difco Laboratories, Detroit, MI, USA) containing 10% horse serum (BHIS) at 37 °C for 24 h. Thereafter, the cultured bacterial cells were centrifuged at 2000×*g* at 4 °C for 15 min and washed twice with saline (0.9% NaCl). Subsequently, the cell pellets were suspended in phosphate-buffered saline (PBS) for in vitro and in vivo experiments.

### Animals

Specific pathogen-free (SPF) 7-week-old female C57BL/6 mice weighing 20–24 g were purchased from Woojungbio (Youngin, Republic of Korea) and maintained at the Woojungbio Testing Facility for 1 week before the start of the experiments. Thereafter, mice were housed under controlled conditions of 50 ± 10% humidity at 23 ± 2 °C and a 12-h light–dark cycle. Mice were fed sterile rodent pellet diet (SAFE A40, Dyets, Bethlehem, PA, USA) and autoclaved sterile water, and there were no restrictions on their diet or water intake. All studies were approved by the Institutional Animal Care and Use Committee of Woojungbio (approval no. IACUC2103-023).

### Design of animal experiments

Mice were randomly allocated to the following seven groups (n = 8 per group): uninfected normal group (Nor), BV-infected control group (Con), GV-infected Metronidazole (100 mg/kg/day)-administered group (Metro), GV-infected low-dose ATCC15009 (1.0 × 10^8^ colony-forming units [CFU]/kg/day)-administered group (15009L), GV-infected high dose ATCC15009 (1.0 × 10^9^ CFU/kg/day)-administered group (15009H), GV-infected low-dose HY7801 (1.0 × 10^8^ CFU/kg/day)-administered group (7801L), and GV-infected high dose HY7801 (1.0 × 10^9^ CFU/kg/day)-administered group (7801H). BV induction by GV infection was performed with slight modifications to a previously established method (Joo et al., 2011). Three days before infection, all mice were subcutaneously injected with 0.5 mg estradiol benzoate (β-estradiol-3-benzoate) dissolved in sesame oil (Sigma-Aldrich, St. Louis, MO, USA) to maintain pseudoestrus. On the day of infection, 20 μL of GV (5.0 × 10^8^ CFU/mL) was inoculated into the vagina of each mouse. Metronidazole and LAB strains were suspended in PBS and orally administered once a day for 7 days from the day after infection. In groups N and C, only saline was administered for the same period. The day after the last administration, mice were sacrificed with carbon dioxide (CO_2_) gas, the vagina was washed with 0.2 mL of sterile saline, and the lavage was collected. After washing, mice were dissected, and vaginal tissues were collected for ELISA and histopathological analysis.

### Detection of GV in mice vaginal lavage

The vaginal lavage was diluted tenfold serially with sterile saline and then plated on Columbia blood agar (CBA, Sigma-Aldrich, St. Louis, MO, USA) containing GV-selective supplement (Oxoid, Basingstoke, UK) and 10% horse blood. After incubating the plate under anaerobic conditions at 37 °C for 48 h, the colonies were counted to measure the number of viable GV, and the results were presented as CFU per 1 mL of vaginal fluid. The abundance of GV in the vagina was measured by quantitative polymerase chain reaction (qPCR) analysis of the ratio of GV-specific DNA to the total bacterial DNA in the vaginal fluid (Happel et al., 2020; Naicker et al., 2021). After determining the number of viable GV, total DNA was isolated (AllPrep Bacterial DNA Kit, Qiagen, Germantown, MD, USA) from the remaining vaginal lavage and qPCR was performed using the QuantStudio 6 Real-Time PCR System (Applied Biosystems, Foster, CA, USA) with GV-specific primer Ba04646236_s1 (Applied Biosystems) and Pan-bacterial primer Ba04230899_s1 (Applied Biosystems).

### Measurement of cytokines in vaginal tissues

The levels of pro-inflammatory (tumor necrosis factor-α [TNF-α], interleukin-1β [IL-1β], and IL-6) and anti-inflammatory (IL-10) cytokines in vaginal tissues were measured with slight modifications to the methods described in previous studies (Choi et al., 2022; Joo et al., 2012). Briefly, 100 mg of vaginal tissue collected from each mouse was homogenized by adding 1 mL of ice-cold radioimmunoprecipitation assay buffer (Thermo Fisher Scientific, Waltham, MA, USA) containing 1% (v/v) protease inhibitor cocktail (Thermo Fisher Scientific). Subsequently, the homogenate was centrifuged at 4000×*g*, 4 °C, for 10 min, and the cytokine concentrations were measured using ELISA kits (BD Biosciences, Franklin Lakes, NJ, USA) according to the manufacturer's instructions.

### Histopathological examination

The isolated vaginal tissues were fixed in 10% neutral formalin for 48 h, then embedded in paraffin and sectioned into 4-μm sections. The sectioned tissues were stained with hematoxylin and eosin (H&E) and analyzed with a digital slide scanner (Motic Instruments, Richmond, Canada). Transitional epithelial and stratum corneum thicknesses were randomly measured at three sites per each tissue in ×100 magnification images (ImageJ version 1.52p, National Institutes of Health, Bethesda, MD, USA). Histopathological analyses including tissue dissection, H&E staining, and image scanning were performed at DooYeol Biotech (Seoul, Republic of Korea).

### Evaluation of LAB viability in a simulated gastrointestinal tract

The digestive stability of LAB strains was assessed by measuring their viability under simulated human gastrointestinal conditions. This experiment was performed with slight modifications to a previously described method (Kim et al., 2022). First, each strain suspended in saline (1.0 × 10^9^ CFU/mL) was poured into a 50-mL conical tube, and 26 μL of 0.3 M CaCl_2_ solution (Sigma-Aldrich) and 4 mL of 6.55 mg/mL α-amylase solution (Sigma-Aldrich) were added. The suspension was then adjusted to pH 7.0 with 1 M NaOH (Sigma-Aldrich) and incubated at 37 °C for 5 min to simulate oral conditions. To simulate gastrointestinal conditions, 6 μL of 0.3 M CaCl_2_, 694 μL of distilled water, and 9.1 mL of 0.07 mg/mL pepsin were added. The pH was adjusted to 3 with 1 M HCl (Sigma-Aldrich) and the mixture was incubated at 37 °C for 1 h. Finally, intestinal conditions were simulated by adding 40 mL of 0.3 M CaCl_2_, 1.31 mL of distilled water, 2.5 mL of 160 mM bile extract, and 16 mL of 22 mg/mL pancreatic solution to the mixture, followed by incubation at 37 °C for 2 h. The survival rate of the strains at each digestion step was determined by measuring the number of viable cells on the MRS plate after aliquoting the mixture of each step.

### Determination of LAB adhesion to HeLa cells

The ability of LAB strains to adhere to the human genital epithelial cells was evaluated by modifying previously reported methods (Choi et al., 2022; Joo et al., 2011; Kim et al., 2021). For this purpose, HeLa, a human cervical epithelial cell line, was purchased from the Korean Cell Line Bank (Seoul, Republic of Korea) and cultured in a 24-well tissue culture plate with 10% heat-inactivated fetal bovine serum (FBS) containing modified Eagle's medium (MEM, Thermo Fisher Scientific) at 37 °C and 5% carbon dioxide (CO_2_). When the HeLa cells reached 100% confluence, the medium was replaced with FBS-free MEM, the LAB strains were inoculated at 1.0 × 10^8^ CFU/well and incubated in 5% CO_2_ at 37 °C for 1 h. After incubation, the plate was washed four times with sterile PBS and treated with 0.05% trypsin–EDTA (Sigma-Aldrich) to detach the cells. HeLa cell counts were obtained using an automated cell counter (Bio-Rad Laboratories, Hercules, CA, USA) and the number of LAB strains was determined.

### Determination of H_2_O_***2***_ and organic acid production

LAB strains were incubated in MRS medium for 24 h at 37 °C in an anaerobic chamber. The whole bacterial cultures (WBC) of each LAB strain were centrifuged at 2,000 × *g*, 4° C for 30 min, and then filtered through a 0.22-μm polyvinylidene fluoride membrane to separate the cell-free supernatant (CFS). Quantification of H_2_O_2_ concentration in the CFS was performed using the OxiTec Hydrogen Peroxide Assay Kit (Biomax, Seoul, Republic of Korea) according to the manufacturer's instructions. Organic acids including lactic acid, acetic acid, butyric acid, and propionic acid were measured using high-performance liquid chromatography (Agilent Technologies, Palo Alto, CA, USA) with an Aminex HPX-87H column (Bio-Rad Laboratories) and a 215-nm UV detector. The mobile phase was 0.001 N H_2_SO_4_ at a flow rate of 0.6 mL/min. Reference materials for the quantification of each organic acid were purchased from Sigma-Aldrich.

### Determination of antibacterial activity against GV

The anti-GV activity of LAB strains was evaluated using WBC and CFS (Choi et al., 2022; Joo et al., 2011). WBC and CFS were prepared with the same procedure used for organic acid analysis described above. GV (1.0 × 10^6^ CFU/mL) was inoculated in the BHIS broth containing 10% (v/v) of each WBC or CFS and cultured under anaerobic conditions for 12 h at 37° C. For controls, 10% (v/v) BHI broth (GV only) or 10% (v/v) MRS broth (GV + MRS) was added instead of WBC and CFS. After incubation, the cultures were serially diluted in sterile saline and then plated on CBA containing GV-selective supplement and 10% horse blood to count GV colonies.

### Evaluation of GV biofilm formation

The inhibitory activity of WBC and CFS against GV biofilm formation was investigated according to the method described in a previous study (Moon et al., 2022). GV (1.0 × 10^7^ CFU/mL) was inoculated into a 24-well high-binding tissue culture plate with 0.9 mL of BHIS broth, and 100 μL of WBC or CFS of each LAB strain was added. The plate was incubated at 37° C in an anaerobic chamber for 24 h, washed with PBS, and 0.1% (v/v) crystal violet solution (Sigma-Aldrich) was added to stain the biofilm. The stained biofilm was washed three times with PBS and air dried for 15 min. After dissolving the biofilm in 600 μL 33% (v/v) acetic acid, the absorbance was measured at 570 nm.

### Determination of GV adhesion inhibition to HeLa Cells

HeLa cells were cultured in 12-well tissue culture plates at 1.0 × 10^6^ cells/mL in MEM supplemented with 10% FBS and 1% antibiotic–antimycotic solution. When the cells reached 90% confluence, the media was replaced with antibiotic-free MEM. GV and LAB strains cultured for 24 h were washed twice in sterile PBS and resuspended in antibiotic-free MEM. For coculture of bacteria and HeLa cells, GV cells (1.0 × 10^7^ CFU/mL) were added to wells and incubated for 2 h in the absence or presence of LAB strains (1 × 10^7^ CFU/mL). After incubation, the cells were washed four times with PBS, detached with sterile distilled water (500 μL), and then diluted in sterile saline. The dilutions were plated on GV-selective CBA to measure the amount of viable GV.

### Effect of HY7801 on GV virulence gene expression

To investigate the underlying mechanism by which HY7801 inhibits GV epithelial cell adhesion and biofilm formation and alleviates GV-induced inflammatory responses, the effect of HY7801 on mRNA expression of GV virulence factors was measured. The cultured GV and LAB strains were centrifuged at 2000×g for 10 min, washed twice with sterile saline, and then resuspended in fresh BHI at 1.0 × 10^7^ CFU/mL. GV and each LAB strain were mixed in a 1:1 ratio and incubated in an anaerobic chamber at 37° C for 3 h. Cocultured GV and LAB strains were centrifuged at 4000×g for 20 min, and total RNA was isolated using the AllPrep Bacterial RNA Kit (Qiagen) according to the manufacturer’s instructions. The cDNA was synthesized using the QuantiTect Reverse Transcription System (Qiagen) and qPCR was performed using PowerTrack SYBR Green Master Mix (Applied Biosystems,) and QuantStudio 6 Real-Time PCR System (Applied Biosystems). Primer sequences of GV virulence factors were designed with reference to a previous study by Qian et al. (Qian et al., 2021), and the details are given in Table 1.

**Table 1:** The primer sequence of *Gardnerella vaginalis* virulence factor genes

Gene name	Sequence	Function
*vly (HMPREF0424_0103)*	F: CTCGCATGCAGTACGATTCT R: TCTGGTGCATCAACGCTTAC	Cytolytic activity
*sld (HMPREF0424_1109)*	F: GGGTTTATGCACACGCTTTT R: GAAAATGCAGACAACGCAGA	Cytolytic activity
*pat (HMPREF0424_0125)*	F: GGTTCTGGCACTATGCTTGG R: ACACGCATTATCCTCCATCC	Epithelial adhesion
*gtf (HMPREF0424_0821)*	F: CAACGAAGGCATAGGTTTCC R: GCGCTTGGAACTGCTTTAAC	Biofilm formation
*atm (HMPREF0424_1253)*	F: ACTTGGCCGTTCACTTTCCA R: AGCCACATACCAACCTGCTC	Metabolism
*stp (HMPREF0424_1297)*	F: TGGCTGTTATTGCTATCTACTTCA R: CTTCCAGAATACTTGCCACTTTGT	Metabolism

### Statistical analyses

Statistical results for in vitro and in vivo animal experiments are presented as mean ± standard deviation at the 95% confidence. Data were statistically compared by one-way analysis of variance and post-hoc Tukey test (multiple groups) or Student’s t-test (two groups) using GraphPad Prism v5 (San Diego, CA, USA).

## Results and discussion

In this study, our primary aim was to evaluate whether HY7801, a LAB known to be effective in treating vulvovaginal candidiasis, could ameliorate BV. Our secondary goal was to elucidate the molecular mechanism by which HY7801 ameliorated BV. To this end, we investigated whether oral administration of HY7801 could alleviate BV using an animal model of vaginal GV infection. In addition, we evaluated the digestive viability, genital epithelial cell adhesion, and antibacterial and biofilm formation inhibitory activity of HY7801 in vitro. Finally, the molecular mechanism of BV inhibition was inferred by analyzing the quantity of H_2_O_2_ and organic acid secretion of HY7801 and the effect of HY7801 on the expression of virulence factor genes in GV.

**Fig. 1:**
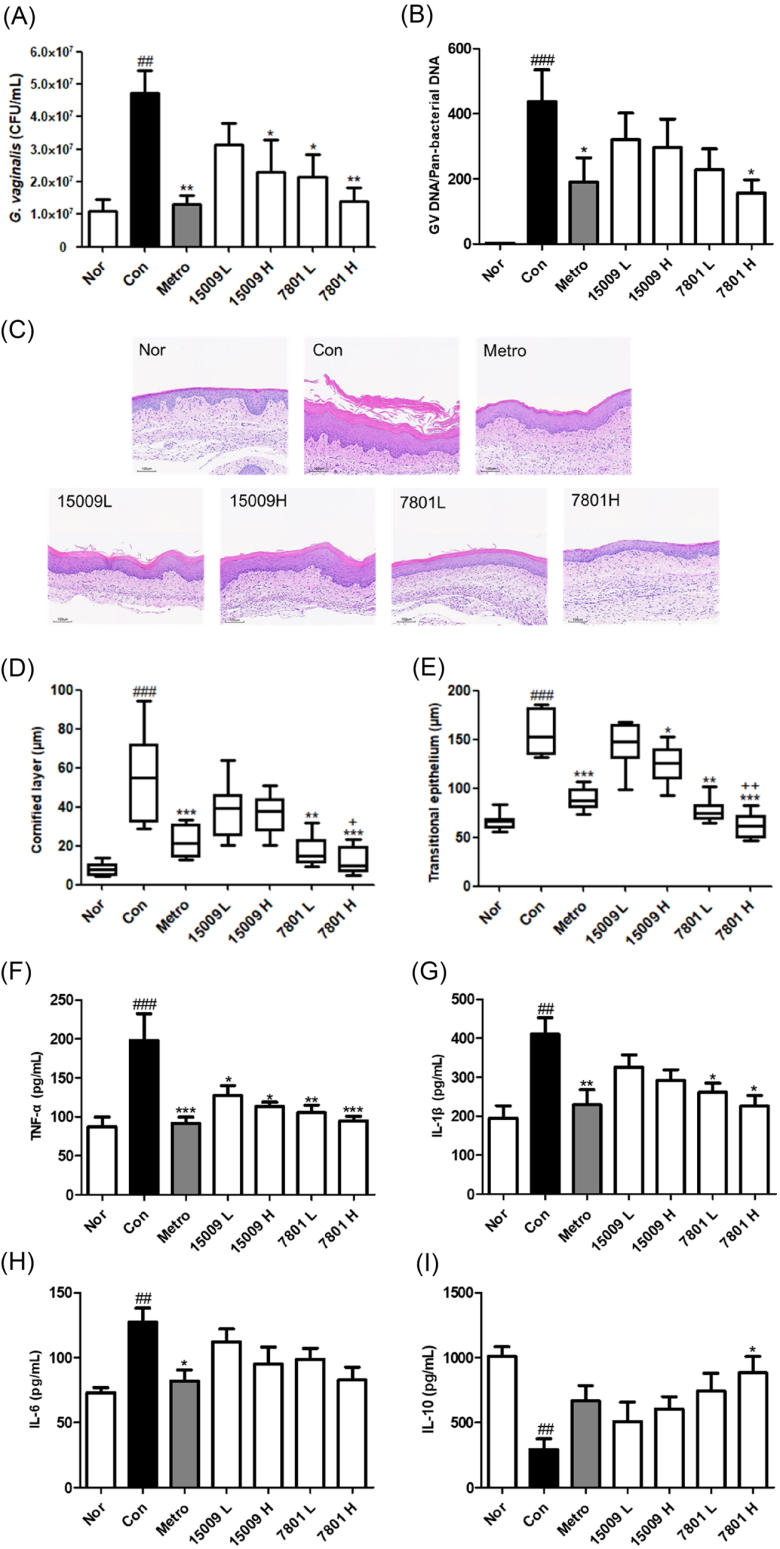
Effect of HY7801 administration on (**A**) the number of viable *Gardnerella vaginalis* (GV) in the vaginal fluid, (**B**) the proportion of GV-specific DNA in vaginal total microbial DNA (**C**) vaginal histological changes (H&E-stained, 100 × magnification), (**D**) vaginal cornified layer thickness, (**E**) transitional epithelium and (**F**, **G**, **H** and **I**) the levels of vaginal cytokines in GV-infected bacterial vaginosis (BV) mice. The results are expressed as the mean ± standard deviation. * *p* < 0.05, ** *p* < 0.01 and *** *p* < 0.001 compared with Con group. ## *p* < 0.01 and ### *p* < 0.001 compared with Nor group. + *p* < 0.05 and +  + *p* < 0.01 compared with 15009H group. *Nor* normal group; *Con* GV-infected no-treatment group; *Metro* GV-infected metronidazole (100 mg/kg)-administered group; *15009L* GV*-*infected low-dose ATCC15009 (1.0 × 10^8^ CFU/kg/day)-administered group; *15009H* GV*-*infected high-dose ATCC15009 (1.0 × 10^9^ CFU/kg/day)-administered group, *7801L* GV*-*infected low-dose HY7801 (1.0 × 10^8^ CFU/kg/day)-administered group, *7801H* GV*-*infected, high-dose HY7801 (1.0 × 10^9^ CFU/kg/day)-administered group

The improvement of BV by HY7801 was evaluated in comparison with ATCC15009, an *L. helveticus* type strain, in vaginal GV-infected BV mouse model. The number of viable GV in the vagina (vaginal lavage fluid) was the highest in the Con group at 4.6 × 10^7^ ± 6.9 × 10^6^ CFU/mL and the lowest in the group that was administered metronidazole, a commonly used antibiotic for the treatment of BV (Metro), at 1.3 × 10^7^ ± 2.7 × 10^6^ CFU/mL except for the Nor group. A decrease in the number of viable GV was observed in all LAB strain-administered groups, showing statistical significance in 15009H, 7801L, and 7801H groups. In particular, the number of viable GV in the 7801H group was the lowest among the LAB-administered groups at 1.4 × 107 ± 4.3 × 106 CFU/mL, which was reduced by 69% compared to the Con group (Fig. 1A). GV-specific DNA present in the vagina was significantly increased in the Con group and decreased significantly in the Metro and 7801H groups. In other treatment groups, such as 15009L, 15009H, and 7801L groups, GV-specific DNA tended to decrease, but this reduction was not statistically significant (Fig. 1B).

Vaginal epithelial cell exfoliation, a major clinical feature of BV, has been reported to be associated with increased transitional epithelial proliferation of the vagina (Bautista et al., 2016; Moon et al., 2022). Therefore, the effect of HY7801 on histological changes of vaginal tissues in GV-infected BV mice was investigated through histopathological analysis. In the vaginal tissues of the Con group, exfoliation of vaginal epithelial cells and an increase in the stratum corneum (cornified layer) and transitional epithelial thickness were observed, whereas in all treatment groups including the Metro group, exfoliation of vaginal epithelial cells and stratum corneum and transitional epithelial thickness tended to decrease (Fig. 1C). Among the LAB-administered groups, 15009H, HY7801L, and HY7801H groups showed significantly lesser increase in cornified layer and transitional epithelial thickness compared with the Con group. In particular, the transition epithelial thickness of the HY7801H group was similar to that of the Nor group (Fig. 1D, E).

Studies have reported that pro-inflammatory cytokines such as TNF-α, IL-1β, IL-6, and IL-8 are elevated in the vagina of women with BV, which may indicate BV severity (Morrill et al., 2020). It was also reported that a decrease in IL-10, an anti-inflammatory cytokine, was observed in animals induced with BV, which could be reversed by treatment of BV (Jang et al., 2017; Joo et al., 2011). The levels of TNF-α, IL-1β, and IL-6 in the vaginal tissues were significantly increased by more than two-fold in the Con group compared with the Nor group. In the Metro group, all pro-inflammatory cytokines were reduced to normal levels. TNF-α was significantly decreased in all LAB-administered groups, whereas IL-1β was significantly decreased only in the HY7801 administration groups (Fig. 1F, G). LAB administration appeared to decrease the level of IL-6, but this decrease was not statistically significant (Fig. 1H). The levels of IL-10 in vaginal tissue were significantly decreased in the Con group and significantly increased in the 7801H group. IL-10 showed a tendency to increase in other LAB-administered groups, but this increase was not statistically significant (Fig. 1I). These results were similar to those of previous studies evaluating the BV inhibitory activity of probiotic lactobacillus strains (Choi et al., 2022; Jang et al., 2017; Joo et al., 2012). Jang et al. suggested that the reduction of inflammatory cytokines and the increase of anti-inflammatory cytokines by probiotics in BV mice may be due to systemic immune response regulation rather than the effect caused by the removal or reduction of vaginal GV (Jang et al., 2017). In our results as well, although the effects of vaginal GV number and vaginal histological changes were different for different LAB strains and doses, inhibition of TNF-α was not significantly different between groups. Therefore, cytokine changes after administration of HY7801 may be the result of systemic immunomodulatory effects. However, this study alone cannot infer all the effects of HY7801 on systemic immunity and additional independent studies are needed to confirm this.

**Fig. 2:**
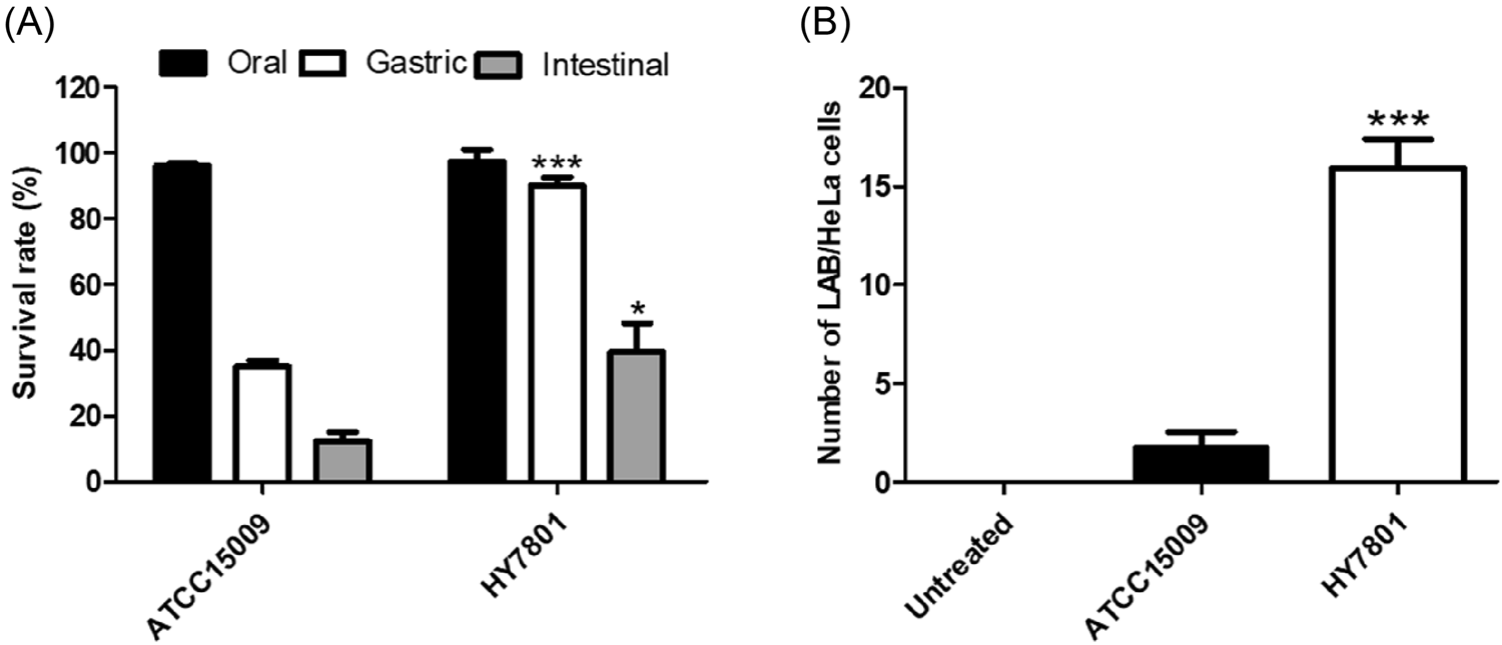
(**A**) Survival rate of lactic acid bacteria (LAB) strains under simulated gastrointestinal tract conditions. (**B**) The number of LAB attached to one HeLa cell under coculture conditions. “Number of LAB/HeLa cells” indicates bacterial colony-forming units (CFU) per HeLa cell count. Data are represented as mean ± standard deviation of three independent experiments. *p < 0.05 and ***p < 0.001 compared with ATCC15009

**Fig. 3:**
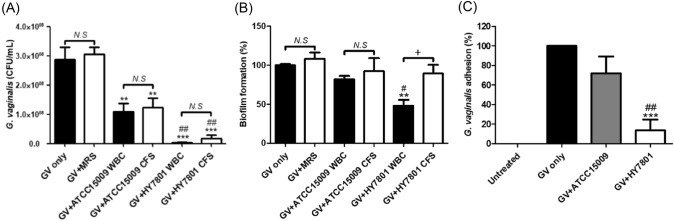
Effects of LAB strains on (**A**) the viability, (**B**) biofilm formation and (C) HeLa cell adhesion ability of GV. Data are represented as mean ± standard deviation of three independent experiments. ** *p* < 0.01, *** *p* < 0.001 and *** *p* < 0.001 compared with GV only. # *p* < 0.05 and ## *p* < 0.01 compared with ATCC15009. + *p* < 0.05 between groups. *NS* not significant, *WBC* whole bacterial cultures, *CFS* cell-free supernatant

**Fig. 4:**
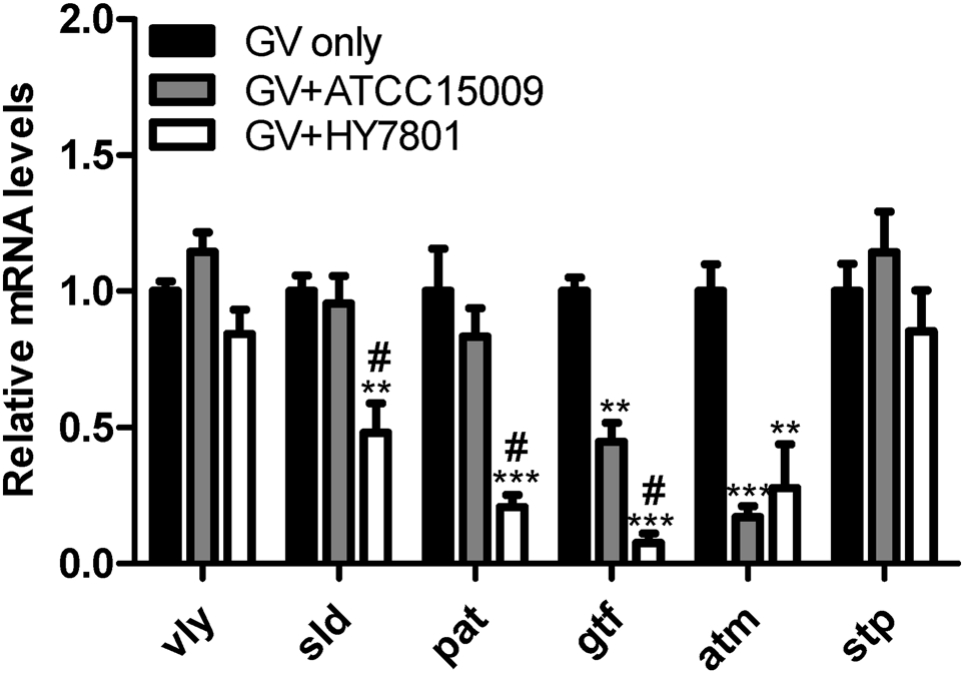
Effects of LAB strains on mRNA levels of virulence-related genes in GV. Data are represented as mean ± standard deviation of three independent experiments. ** *p* < 0.001, *** *p* < 0.001 compared with GV only. #*p* < 0.05 compared with ATCC15009

Orally administered probiotics, especially *Lactobacillus* spp., can reach the vagina through the GIT and attach to vaginal epithelial cells to secrete organic acids and H_2_O_2_ to suppress colonization and growth of pathogenic bacteria (Liu et al., 2020; Reid and Bruce, 2003). Therefore, digestive viability, epithelial cell adhesion, and the ability to secrete H_2_O_2_ and organic acids can be major requirements for determining probiotic strains for BV prevention and treatment (Choi et al., 2022; Vásquez et al., 2009). In a preliminary experiment, it was confirmed that HY7801 showed the best adhesion ability to genital epithelial cells (Figure S1) and lactic acid and H_2_O_2_ secretion ability among approximately 25 human-derived probiotic LAB (Table S1). In an in vitro test using *L. helveticus* type strain ATCC15009 as a reference strain, it was confirmed that HY7801 showed superior GIT viability (Fig. 2A) and HeLa cell adhesion compared to the type strain (Fig. 2B). In addition, HY7801 showed significantly higher antibacterial activity against GV than the type strain (Fig. 3A) due to its ability to generate high H_2_O_2_ and lactic acid during growth (Table 2). When the pH was adjusted to 7.0 with NaOH or H_2_O_2_ was neutralized by catalase treatment, the antibacterial activity of HY7801 was reduced (Figure S2). Therefore, it was determined that the growth inhibition and antibacterial activity of HY7801 against GV was a synergistic effect of H_2_O_2_ and lactic acid. It is known that GV in the vaginal epithelium forms a biofilm to provide a favorable environment for the proliferation of other pathogenic bacteria, which can interfere with antibiotic treatment (Castro and Cerca, 2015; Swidsinski et al., 2011). In addition, it has been reported that GV promotes persistence of BV by producing virulence factors such as vaginolysin (*vly*) and sialidase (*sld*) that interfere with the immune response of the host (Lewis et al., 2012; Qian et al., 2021). In an in vitro experiment of co-culture of HY7801 and GV, HY7801 inhibited GV adhesion to HeLa cells by more than 80% (Fig. 3C), and WBC of HY7801 effectively inhibited GV biofilm formation (Fig. 3B). Interestingly, the CFS of HY7801 did not significantly inhibit GV biofilm formation (Fig. 3B), unlike previous studies that showed that the CFS of specific probiotics inhibited GV biofilm formation (Moon et al., 2022; Qian et al., 2021). Therefore, in order to exclude the effects of lactic acid and H_2_O_2_, GV and HY7801 were co-cultured in fresh media, and the expression of GV virulence factor genes related to immune avoidance, epithelial cell attachment, biofilm formation, and energy metabolism was confirmed. HY7801 significantly reduced the mRNA levels of *sld, pat,* and *gtf*, which are involved in sialidase secretion, vaginal epithelial cell adhesion, and biofilm formation, respectively, in GV compared with the type strain (Fig. 4). This suggests that the effect of HY7801 on the BV animal model may be due to its antibacterial activity against GV as well as HY7801 directly regulating the virulence factor gene of GV. Sabbatini et al. reported that probiotics such as *Saccharomyces cerevisiae* CNCM I-3856 and *Lacticaseibacillus rhamnosus* ATCC 53,103 inhibit GV adhesion to epithelial cells and biofilm formation by co-aggregation of GV (Sabbatini et al., 2020). It is thought that there is a relationship between the ability of probiotics to co-aggregate GV and to regulate expression of virulence genes, which is worth considering for future studies.

**Table 2:** Organic acids and H_2_O_2_ production (μg/mL) profile of the LAB strains

Strain	H_2_O_2_	Lactic acid	Butyric acid	Acetic acid	Propionic acid
ATCC15009	1.20 ± 0.09	782.77 ± 3.34	ND	1,458.55 ± 2.92	ND
HY7801	3.53 ± 0.10*	1,218.70 ± 32.2*	ND	1,286.46 ± 13.13	ND

Data are represented as mean ± standard deviation of three independent experiments

*ND* not detected

^*^p < 0.05 compared with ATCC15009

It was demonstrated in this study that HY7801 effectively ameliorated GV-induced BV pathologically and immunologically in animals. In addition, it was found that the effect of HY7801 was due to its excellent GIT viability, antibacterial activity, and GV virulence factor gene expression regulating activity. Therefore, HY7801 can be proposed as a probiotic to improve BV symptoms caused by GV infection. Currently, we are planning a clinical trial to validate the effect of HY7801 on vaginal microflora imbalance. Nevertheless, the limitations of this study are that only a single strain of GV was used as a model for BV and the effect of HY7801 on systemic immunity could not be evaluated. Therefore, confirmation of antimicrobial efficacy against various urogenital related pathogens such as *Prevotella bivia* and *Atopobium vaginae* may be necessary, and further studies are needed to elucidate the molecular communication mechanism between HY7801 and the host immune system.

## Supplementary Information

Below is the link to the electronic supplementary material.

Electronic supplementary material 1 (DOCX 83 kb)
