# Supplementary material for: Lactobacillus helveticus HY7801 ameliorates bacterial vaginosis by inhibiting biofilm formation and epithelial cell adhesion of Gardnerella vaginalis
Source: Food Sci Biotechnol. 2022 Dec 1;32(4):507–15. doi: 10.1007/s10068-022-01208-7 (PMC9992491; doi:10.1007/s10068-022-01208-7)
Supplement: Supplementary file 1 — Electronic supplementary material 1 (DOCX 83 kb) [file 10068_2022_1208_MOESM1_ESM.docx]

**Supplementary Materials:**

**Materials and methods**

**LAB strains**

*L. acidophilus* (LA04, LA351, LA353, LA595, LA613), *L. brevis* LB74, L. gasseri (HLAB4-14, L18, L141204, L307) *L. helveticus* (HY7801, LH596, LH597, LH599, LH151725), *L. johnsonii* HY7042, *L. reuteri* (MBM4-4, LMB8-2, LMB5-5) and *Lactobacillus* sp.( KM21-1, KM5-5, KY16-11, L260, L266) were stored in seed cell library at hy Co., Ltd. (Yongin, South Korea). *L. rhamnosus* GR-1 was purchased from the American Type Culture Collection (ATCC), and *L. reuteri* RC-14 was isolated from a commercial probiotic product (Chr. Hansen, HQ, Denmark).

**Cell Lines**

HeLa, a human cervical epithelial cell line, and A431, a human vulvar epithelial cell line, were purchased from the Korean Cell Line Bank (Seoul, Republic of Korea) and cultured in modified Eagle's medium (MEM, Thermo Fisher Scientific) containing 10% FBS at 37 °C and 5% CO_2_.

**Measurement of genital epithelial cell adhesion ability of LAB strains**

The adhesion rates of LAB strains to HeLa cells and A431 cells were measured by modifying the method of Jacobsen et al. ([Jacobsen et al., 1999](#_ENREF_1)). The LAB strains suspended at a concentration of 1.0 × 10^8^ CFU/mL (A0, CFU/mL) in FBS-free MEM were treated with HeLa cells or A431 cells cultured in a 6-well plate and incubated for 2 hours in CO_2_ incubator. After incubation, the plates were washed 3 times with PBS and treated with 0.05% trypsin-EDTA for 5 minutes to separate cells. The isolated cells were serially diluted with sterile saline and inoculated on MRS agar plates. The plates were incubated at 37 °C for 72 h to count colonies (A1, CFU/mL). The adhesion rate was calculated as follows.

% adhesion ability = (A1/A0) × 100

**Comparison of hydrogen peroxide production ability of LAB strains**

The hydrogen peroxide-producing ability of LAB strains was qualitatively measured on MRS-TMB agar plates with reference to a study by Rabe LK et al. ([Rabe and Hillier, 2003](#_ENREF_2)). Briefly LAB strains were inoculated on MRS agar plate containing 0.025% Tetramethylbenzi (TMB), 0.005% Hemin, and 0.001% peroxidase and incubated anaerobically at 37°C for 48 h. After incubation, the plates were exposed to air to induce color development. If the color of the colony does not change to blue within 30 minutes after developing the color, there is no production (-), if only a part changes, it produces a part (+), if the whole colony is blue, it produces (++), if the whole colony is dark blue, it produces high hydrogen peroxide (+++) was considered.

**Effect of pH adjustment and catalase treatment on antibacterial activity**

To evaluate the effect of pH and catalase on antibacterial activity of the HY7801 CFS against *G. vaginalis*, pH of the CFS was adjusted to 3.0, 5.0, 7.0 and 9.0 with 5 M NaOH. The CFS was also treated separately with catalase (Sigma-Aldrich) at a final concentration of 1 mg/mL. Untreated CFS was included as a control whereas MRS broth (uncultured media) was used as a negative control. *G. vaginalis* was inoculated at 1% (v/v) in 96-well plates with BHIS broth containing 5% (v/v) CFS with different pH or treated with catalase. The plate was incubated at 37 °C for 24 h under anaerobic condition and its OD was measured at 600 nm.

**Figures**


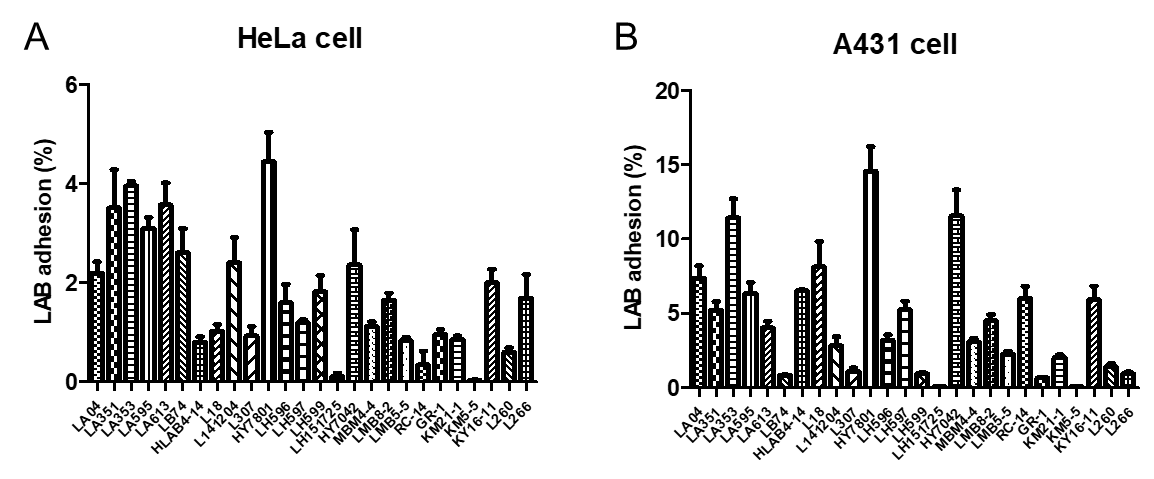


**Figure S1**: Genital epithelial cell adhesion ability of LAB strains on (A) HeLa cells and (B) A431 cells. The LAB adhesion rate was calculated as the ratio of the number of epithelial cell-attached bacteria to the number of treated bacteria. Data are represented as mean ± standard deviation of two independent experiments


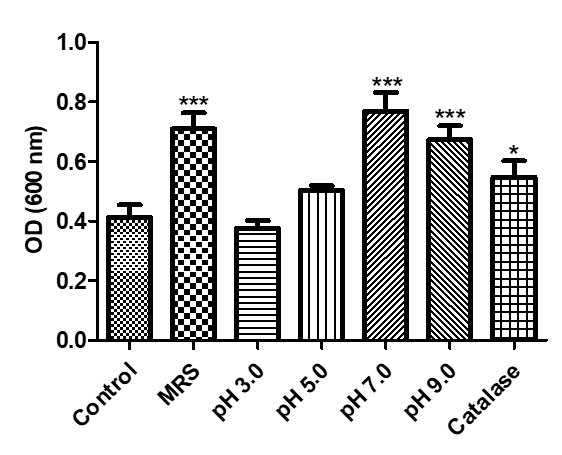


**Figure S2**: Optical density (OD) of *G. vaginalis* suspension incubated for 24 h with 5% (v/v) HY7801 cell-free supernatant treated at different pH or catalase. Data are represented as mean ± standard deviation of three independent experiments. * *p* < 0.05 and *** *p* < 0.001 compared with Control (untreated CFS).

Table S1. Confirmation of H_2_O_2_ production of LAB strains in TMB contained MRS plate

| **No.** | **Species** | **Strain** | **H_2_O_2_ production** |
| --- | --- | --- | --- |
| 1 | *L. acidophilus* | LA04 | ++ |
| 2 | *L. acidophilus* | LA351 | + |
| 3 | *L. acidophilus* | LA353 | + |
| 4 | *L. acidophilus* | LA595 | ++ |
| 5 | *L. acidophilus* | LA613 | + |
| 6 | *L. brevis* | LB74 | ++ |
| 7 | *L. gasseri* | HLAB4-14 | ++ |
| 8 | *L. gasseri* | L18 | ++ |
| 9 | *L. gasseri* | L141204 | + |
| 10 | *L. gasseri* | L307 | + |
| 11 | *L. helveticus* | HY7801 | +++ |
| 12 | *L. helveticus* | LH596 | + |
| 13 | *L. helveticus* | LH597 | ++ |
| 14 | *L. helveticus* | LH599 | + |
| 15 | *L. helveticus* | LH151725 | - |
| 16 | *L. johnsonii* | HY7042 | ++ |
| 17 | *L. reuteri* | MBM4-4 | + |
| 18 | *L. reuteri* | LMB8-2 | + |
| 19 | *L. reuteri* | LMB5-5 | + |
| 20 | *L. reuteri* | RC-14 | + |
| 21 | *L. rhamnosus* | GR-1 | + |
| 22 | *Lactobacillus sp.* | KM21-1 | + |
| 23 | *Lactobacillus sp.* | KM5-5 | + |
| 24 | *Lactobacillus sp.* | KY16-11 | ++ |
| 25 | *Lactobacillus sp.* | L260 | - |
| 26 | *Lactobacillus sp.* | L266 | - |

(-), No production, (+), H_2_O_2_ partial production, (++), H_2_O_2_ production, (+++), H_2_O_2_ high production

**References**

Jacobsen CN, Rosenfeldt Nielsen V, Hayford AE, Moller PL, Michaelsen KF, Paerregaard A, Sandstrom B, Tvede M, Jakobsen M. Screening of probiotic activities of forty-seven strains of Lactobacillus spp. by in vitro techniques and evaluation of the colonization ability of five selected strains in humans. Appl Environ Microbiol 65: 4949-56 (1999)

Rabe LK, Hillier SL. Optimization of media for detection of hydrogen peroxide production by Lactobacillus species. J Clin Microbiol 41: 3260-4 (2003)
